# From Our Correspondents

**Published:** 1988-11

**Authors:** 


### From our Correspondents

**Integration of General Medicine—Geriatrics**

The South West is very much to the fore in the establishment of joint appointments in Care of the Elderly and General Medicine. This move, supported by the College, is slowly making headway in the country as a whole, with some regions more actively developing this approach than others. At the time of writing it looks as if there may well be three new combined appointments of this type in the South West in the not too distant future, with a fourth a few years away. Although I can understand and accept the arguments of those who prefer the principle of a separate autonomous Department of Geriatric Medicine, and appreciate the valuable contribution they make to health services for the elderly, it seems clear that the current demographic trend is going to be more and more difficult to cope with if we stick to specialist autonomous departments. Another encouraging development is the increasing involvment of Departments of Geriatric Medicine in other specialities where many older people tend to be admitted, e.g. orthopaedics. It is essential that our expertise and knowledge is made increasingly available in such areas, not just for the benefit of our colleagues but particularly to service the needs of the increasing numbers of elderly people they are going to have a treat. In my view this argument should be a strong plank in any case of need for additional manpower, at whatever level in Departments of Geriatric Medicine, whether integrated with General Medicine or not.

**Alzheimer's Disease**

Changing now to another subject—THA (tetrahydroamino-acridine). Is this anticholinesterase the first step towards a rational therapy for sufferers with Alzheimer's disease? After the early report by Summers in November 1986, many centres have been working with this substance. The main draw-back appears to be its hepatotoxicity and cholinergic agonist peripheral side effects. Some trials have had to be temporarily halted because of the hepatotoxicity, but most are now back in full swing. Some quite dramatic results have been claimed at a purely anecdotal level but in my opinion although THA appears to be helpful to a certain extent in some subjects, it is not going to prove as effective as say, levodopa, in Parkinson's disease. Nevertheless, it looks as if we may at last have our foot on the first rung of the ladder. It is also important to remember that Summers' original trial, and the majority of the others, included lecithin as well as THA, and it may be wrong to think of THA alone as an indicator of future therapeutic development.

G. K. Wilcock

**Professor John Farndon**

The occasion of the South West Surgeon's Club Meeting at Barnstaple gave me an opportunity to sit in the sunshine outside the Saunton Sands Hotel watching the surf breaking along the huge expanse of Barnstaple Bay and talk to John Farndon, the newly appointed Professor of Surgery at Bristol University whom I had only recently met, and to ask him about his career, his ideas and his plans for the future.

He felt his life as dividing itself into three phases, his boyhood and his schooling in Sheffield, his medical education and his surgical training at Newcastle University and Hospitals, where until recently he was Senior Lecturer in surgery under Professor Ivan Johnston and now he is entering into phase 3 with his appointment to the Chair of surgery at Bristol. He spoke of this as though he meant it to be his culminating phase, which is good news to us whose last two professors have moved on to what they imagined would be greener pastures. Along the road he has had two sojourns in the USA, the first for two years, in 1979–81 as Peel Travelling Scholar to Duke University, at Durham, North Carolina, working with Sabiston and Wells on Endocrine surgery. The second in 1984, as James IV Travelling Scholar, visiting centres specialising in Endocrine surgery. In 1986 he was appointed to a Hunterian Professorship at the Royal College of Surgeons and gave his lecture on Parathyroid surgery.

His special interests include breast disease and endocrine surgery. I asked him his views on the National Breast Screening programme which is now being set up. He regards this, without much enthusiasm, as an inevitable development. He thinks that it must be very rigidly controlled and he is sceptical that it will have much effect on the longevity of breast cancer sufferers. It will be expensive in medical resources and in emotional trauma to that whole group of women who will be exposed to it. There will be high expectations and perhaps much litigation. If it does not yield real benefit the fact must be faced and the breast screening machinery closed down.

He sees the West of England as a region in which Bristol Medical School should play an important part, there is a wealth of clinical material and of clinical expertise here and he would like to encourage student attachments for some period in their final year when they could really benefit from it. Of course students like their elective jaunts around the Third World but he feels that they should go later when they themselves are qualified and have some real experience to contribute.

He is worried about the future of manpower allocation and feels it is often constrained by wrong pressures. He fears the power of pressure groups to divert resources from essentials.

Turning to his plans for his own department he wants it to be broadly based and not to become too specialised. His main research effort will go into work he has been pursuing in Newcastle for some time on breast cancer. He believes that variations in surgical and radiotherapeutic techniques will yield little in the future and that hormonal manipulations have been fully exploited. He thinks that progress may be made by better understanding of the biology of tumours, for example, the detection of tumours expressing epidermal growth factor receptors segregates a group of ladies with tumours associated with a very poor prognosis. In vitro testing can be done on various chemotherapeutic agents to determine tumour sensitivity, as bacteria are tested for antibiotic sensitivity. It may be possible to make these agents 'home in' on tumour cells in the manner of Ehrlich's 'magic bullet' using the cell surface receptor.

His sport is squash and occasional golf. He enjoys physical work in the open air, in the garden and on the farm. Music is a great joy to him, all his family are musical and play a variety of instruments. He plays the recorder "badly" but his chief pleasure is listening to recorded music with the score. Twentieth century music makes a great appeal to him, particularly the British composers, Britten, Walton and Vaughan Williams. Martinu and Janacek are other favourites.

I shall remember for a long time the pleasure of sitting in the sunshine enjoying the easy flow of conversation on these and indeed all subjects with this friendly fellow Yorkshireman. I think he will do very well for Bristol and for the West Country.

M. G. Wilson

**Medicine and Retirement**

Increasing numbers of doctors are retiring before the age of sixty-five, and there are financial inducements to do so from The National Health Service. Some who retire wish to give up their careers completely in order to live abroad, or pursue hobbies such as gardening or sailing, or change careers in order to write, teach or join the Church. Many doctors serve on charitable bodies of various kinds. Others miss the challenge of medicine in the N.H.S. and take up full-time professional work in other countries, especially those which are less well developed. Alternatively, they do regular locum or assistant posts in this country. The majority of doctors who retire prefer to run down the pressure of work gradually. They take up insurance work, or advisory posts in industry, or act as medical officers to companies; they may act as assessors for mobility allowance or serve on medical boards or tribunals. Others continue to care for their private patients, many of whom become devoted to an individual practitioner.

Most retired doctors I have met seem happy–they have decided how much work they wish to do and are relieved of the pressure which their previous practice imposed on them. There is universal delight when they receive the letter from their defence organisation telling them that they no longer pay the subscriptions! So many of my retired colleagues feel their lives have become more balanced and full, thanks to the increased time they can devote to hobbies such as painting, D.I.Y., horticulture, music, golf or travel, to name just a few. We are indeed fortunate in our profession that pension arrangements allow us to enjoy retirement years in comfort.

Non-medical friends talk to me more freely these days (or is it that I have more time to listen?). There are three common themes. One is that they feel our profession complains too much about being over-worked and being subject to poor facilities and cuts. They find this incompatible with the frequent difficulties they have in getting hold of their doctor, and the overt signs of government investment in the N.H.S.—Southmead is like a huge building site! Another criticism I hear frequently is the inordinate delay in clinics and accident departments which could often be relieved by improved organisation. A recent example was told me of a clinic where all the patients are booked in at the same time, and by the time X-rays are done, it is often 4 to 5 hours before they get away. The third recurring criticism of our profession is the failure of doctors and nurses to explain things. In an increasingly technological age, we can investigate and treat diseases much more accurately than ever before, but we still need to find time to listen to patients' problems carefully and then explain the situation to them. Many people, especially the elderly, do not want high-tech procedures unless they are confident that there will be clear benefit. This failure of many of us to communicate with our patients is responsible for much dissatisfaction which can end in some form of complacent procedure, or even litigation.

H. G. Mather

**Auberon Waugh on the Imprisonment of a Bristol Doctor**

With the permission of the Sunday Telegraph we reprint a passage from Auberon Waugh's column on Sunday 18th September. He is concerned that inappropriate sentencing is exacerbating the problem of overcrowded prisons. He says "With the desperate need for prison space it is particularly irritating to read of people being sent to prison whose offence would much better be punished by a hefty fine. Recently a Bristol doctor was given two years for supplying drugs to a massage parlour girl, already a heroin addict, in exchange for sexual services. A bad thing to do we must agree, but what possible advantage is there to the community in sending him to prison for two years rather than fining him £10,000." He might have added that he had already suffered the severest punishment with inevitable removal from the medical register and the loss of his professional livelihood. To many colleagues he was a charming, cultured and sensitive man whose lapse seems incomprehensible. His prison sentence only deepens his personal tragedy.

M. G. Wilson

**Inaugural Lecture in General Practice**

The first Inaugural Lecture by a Professor of General Practice in the South Western Region was delivered at 5.15 pm on Tuesday 28 June 1988 by Professor Denis Pereira Gray from the Department of General Practice and the Postgraduate Medical School of the University of Exeter.

The first academic appointment of a General Practitioner within the Region was at Exeter on 1 December 1973 and the Department there has grown over the years. Professor Gray was appointed to his Chair on 1 October 1986 and it is thought to be the first Chair of General Practice in a Postgraduate Department in the British Isles.

The lecture, entitled 'Families and their Family Doctors' was in five parts, the first being an introduction and acknowledgement to the many friends and colleagues who had contributed to the academic development of general practice within the Region. Professor Gray paid particular tribute to the University of Exeter, the Health service Authorities in the South Western Region and the profession itself which had always given quite unusual support.

In the second part Professor Gray outlined the nature of the discipline of general practice and its place within the University world. He then went on to emphasise the particular importance of research, both as a means of identifying new knowledge and of research methods of training and thinking.

In the fourth part of the lecture he reported some research he had himself undertaken on the characteristics of those with some forms of family history and concluded by speculating on the future development of medicine and society and foresaw an increasing role for the general practitioner in health care in the future.

**The Bristol Medico-Historical Society**

A note appeared in this journal in January 1986 with the proposal to form a society of individuals sufficiently interested in medical history to be prepared to give a paper themselves to the society if formed. A dozen or so initial volunteers got the idea off to a good start and now we have our optimum number of two dozen. Professor John Clamp is the first president and the undersigned the first secretary. With three meetings a year and two papers given at each meeting, a member is required to give a paper once in four years, this four year cycle can also signal a change of President and Secretary. The meetings take place at Frenchay Postgraduate Centre where also is the Monica Britton Museum of Medical History, and are preceded by a dinner. Any number of guests can be invited. So far we have had papers on 'The discovery of Bence-Jones Protein', 'Hippocrates and the Aesculapeum at Cos'. 'The Mariner of Bristol', 'The Royal Malady', 'The Museum Mummy', 'The care of the newborn in antiquity', 'Golla, Grey and the little green screen', 'A Cotswold pioneer anaesthetist', 'Medical services in Weston super Mare', 'Archibald Menzies, a surgeon botanist of the 18th century', 'Surgical Fellows of the Royal Society', 'Childbirth — lessons from the past'. 'Caleb Hillier Parry', 'American Prisoners of War in Dartmoor 1813–1815'. A visit was arranged to the Royal Library at Windsor Castle to see the Leonardo Anatomical Drawings and another is planned to the Royal College of Surgeons to see the Hunterian Collection. The programme for the year ahead has already been volunteered. We seem to have a formula for some very enjoyable and informative meetings, the secretary has no trouble in getting speakers, the speakers have no trouble in attracting an audience. We all have a stimulus to work up a subject of special interest to us and the pleasure of discussing it with an intimate group of friends.

M. G. Wilson

**250 years of In-patient Care at Bristol Royal Infirmary**

During the weekend of 12th/13th December 1987 a symposium was organised to mark 250 years of patient care at the B.R.I. A social programme was also arranged for visitors, and the Nurses' League was asked to provide 'hostesses' as escorts for these visitors, mainly doctors wives. We met at the Jenner Centre at 9.30 a.m. and after introductions all round (people came from all over the country), we boarded the coach provided, where our official guide for the day, Mr Ron Snook, was waiting and whose commentary throughout the day was excellent.

Our first visit was to Quakers Friars, to see the Bristol Tapestry displayed in all its glory. If you have not yet seen this, do go, it is well worth a visit and a good browse. It encapsulates Bristol's history in a fascinating and unusual way. In addition one of our members, Milly Painton, worked on a large section of it.

While at Quakers Friars we saw William Penn's marriage certificate and other documents, and looked at the Planning Exhibition.

From there, we were taken with anecdotes and comments from Mr Snook to places of interest, to the Theatre Royal, where a member of the management gave us a talk on the history of our oldest working theatre — of necessity mainly the highlights, or we would have been there all day, there is so much to tell. We were allowed on-stage amid the set for "Mr. Toad",—backstage and into part of the workshops and also into the New Vic. We did *not* see the ghost of Sarah Siddons who, I believe, is said to appear now and then—just as well, perhaps, but we did enjoy our coffee there.

Another short trip took us to St. Mary Redcliffe Church and we were lucky enough to have Cannon David Frayne to show us round, ably assisted by the verger. It is a beautiful church, again, so packed with Bristol's history, that I could not possibly do it justice in a short account. Some of the treasures, apart from the building itself and its chapels, were some lovely hand-worked altar embroideries, and as a special privilege, the cope chest was unlocked—a fascinating piece of furniture in itself—and we were shown some of the elaborately embroidered copes worn by the clergy—one or two still in use.

After a very good buffet lunch at the Jenner Centre, and as maybe imagined a good chat, we embarked again for our last visit—to the S.S. Great Britain. Fortunately the weather was kind, a video gave us a potted history and a guide was eloquent. As one who, with my family, watched the return of the "hulk" up-river in the early 70s, it is marvellous to see how much restoration has been achieved and one can imagine our Victorian forbears promenading on the decks.

After a cup of tea at the Unicorn Hotel the day was ended. Everyone seemed to agree that it had been a most interesting and informative day. Bristol has a long and colourful history about which I thought I knew quite a lot, but discovered that there is always twice as much to learn.

The visitors returned to the Grand Hotel where they had a Gala dinner in the evening, and on Sunday a tour of Bristol Cathedral, followed by Evensong. I hope the doctors enjoyed their day as much as we did—incidentally some of the subjects for their lectures looked most interesting and I would not have minded being a "fly on the wall".

Altogether it seemed to be a very successful weekend.

Peggy Young

**Overnight Sleepers**

This harmless little piece was written on a delayed overnight train from Edinburgh to Bristol but might infuriate many people. I certainly haven't complained to British Rail, but am vaguely aware that to congratulate them could be open to misinterpretation. It depends on your point of view.

Travel is often a curious experience. It seems to lead to all sorts of literary effusions. To be honest I don't know how you, gentle reader, get about, or even if you like travel.

How to go about it? Car, bus, air or boat for a start. No — not for me; given the choice it can only be by train. Yes, I know why. It is the long-term ill effect of being a schoolboy in grey shorts haunting the G.W.R. sheds at Willesden whilst of an age when crossing bare railway lines held the sort of thrill that saying something outrageous at a so-called serious meeting has now. Then, as schoolboy trespasser my real ambition was not really to be a locomotive driver, but to travel on the luxurious and shiny Pullman sleepers. They always looked so impressive in the stations — and best of all at Victoria. Nice chocolate and cream colours together with a stylish moulded shapeliness.

So now in middle age one can—more or less — enjoy the same anticipated pleasures. Even so *Inter-City* doesn't quite do what seemed possible in the 1950s. Reliability, time saved, lack of effort seem to be the watch words in the current British Rail advertising gamuts. Whether this projected image is really true or not is perhaps a moot point. However, it is the next best thing to the old image of railways and the Pullman style. Why, you even have the comfort of driving on the left hand side. For a train enthusiast, all this modern image projection is irrelevant. The ambition remains the same—the farther the better.

Our usual day-to-day sort of time in doing this has no meaning. The aim of a traveller is all rather like wanting to be back in the Middle East again. Ordinary time really has no meaning unless you are quite incapable of enjoying travel purely for its own sake. If the time of arrival is essential, then it is best not to travel.

To me the pinnacle of enjoyment of rail travel is achieved at night. For a start you are not distracted by the details of what is going on outside. Fishermen will understand this approach—or at least some of them. In that field it is commonplace to remark casually to spouse or children that there is 'more to fishing than catching'. An ancient, if eccentric Chinese Emperor once carried this philosophy to an understandable extreme. He must indeed have had very inquisitive children, wives, relatives and friends. He evidently fished with great enthusiasm, but without the inconvenience of a hook at the end of his line.

Well done. As usual the Chinese have beaten us to it. Non-catching fishing is just like late-arriving travel. We Europeans really are thick. It is all a question of confusing means with aims. Modern management can't understand this difference either.

Now back to the original point. What is the sense in using an overnight sleeper?

In my view it is the acme of travel.

For a start though, it must be—sadly—a first class one. It is only there that one can indulge in ridiculous thoughts without the need to worry about the deplorable insomniac habits of one's companion. Who knows, worse still he might think that a sleeper was for sleeping in. Good travelling.

J. D. Davies

